# Interdisziplinäre ultraschallgesteuerte minimal-invasive Autopsie bei COVID-19-Verstorbenen auf der Intensivstation einer Universitätsklinik: Eine Proof-of-Concept-Studie

**DOI:** 10.1007/s00292-023-01248-8

**Published:** 2023-12-05

**Authors:** T. Lahmer, K. Stock, S. Rasch, S. Porubsky, S. Jeske, C. Schustetter, U. Protzer, U. Heemann, R. Schmid, W. Weichert, G. Weirich, J. Slotta-Huspenina

**Affiliations:** 1 Klinik und Poliklinik für Innere Medizin II, Klinikum Rechts der Isar, Technische Universität München, München, Deutschland; 2 Abteilung für Nephrologie – Nierenheilkunde, Klinikum Rechts der Isar, Technische Universität München, München, Deutschland; 3 Institut für allgemeine Pathologie, Johannes Gutenberg-Universität Mainz, Mainz, Deutschland; 4 Institut für Virologie, Technische Universität München, München, Deutschland; 5 Institut für Allgemeine Pathologie und Pathologische Anatomie, TUM School of Medicine and Health, Technische Universität München, München, Deutschland Trogerstr. 18, 81675; 6 Pathologie Starnberg MVZ GmbH, Starnberg, Deutschland

**Keywords:** Postmortale Biopsie, Gewebesampling, Multidisziplinär, Postmortales Intervall, Nadelbiopsie, Postmortem-biopsy, Minimally invasive tissue sampling, Multidisciplinary, MIA, MITS

## Abstract

In dieser Machbarkeitsstudie führten wir in einem interdisziplinären Team standardisierte ultraschallgesteuerte minimal-invasive Autopsien (US-MIA) unmittelbar am Krankenbett von COVID-19-Verstorbenen auf der Intensivstation des Klinikums Rechts der Isar der Technischen Universität München (TUM) durch. Die Studie hatte zum Ziel, Machbarkeit, zeitliche Effizienz und infektionshygienische Aspekte des Verfahrens sowie die Qualität der Gewebeproben zu überprüfen. Unsere Ergebnisse zeigen, dass die bettseitige US-MIA geeignet ist, Gewebeproben vor Einsetzen der postmortalen Autolyse zu gewinnen, und dass sie zudem schnell und sicher durchgeführt werden kann. Das bisher wenig beachtete Potenzial der US-MIA verdient besondere Aufmerksamkeit im Kontext der postmortalen Diagnostik, Forschung und Qualitätssicherung. In Zukunft könnten diese Stärken der US-MIA dazu beitragen, die postmortale Diagnostik in die Moderne der pathologischen Tiefenanalytik („Omics“) zu führen.

## Abstract (en)

In this feasibility study, we carried out in an interdisciplinary team standardised, ultrasound-guided, minimally invasive autopsy (US-MIA) directly at the bedside of patients who died of COVID-19 in the intensive care unit of the Rechts der Isar Hospital of the Technical University Munich (TUM). The aim of the study was to verify the feasibility, time efficiency and infection hygiene aspects of the process, as well as the quality of the tissue samples. Our results show that bedside US-MIA is suitable for obtaining tissue samples before the onset of postmortem autolysis, and that it can also be carried out quickly and safely. The potential of US-MIA, which has gained little recognition so far, deserves special attention in the context of postmortem diagnosis, research and quality assurance. In the future, these strengths of US-MIA could help to lead postmortem diagnosis into the modern age of pathological deep analytics (“omics”).

Obwohl die konventionelle Autopsie nach wie vor als Goldstandard der fachübergreifenden Qualitätssicherung in der klinischen Medizin gilt, beobachten wir seit Jahrzehnten einen rückläufigen Trend bei klinischen Autopsien. Die Gründe dafür sind vielfältig. Aufgrund der Erweiterung des diagnostischen Spektrums durch verbesserte und weit verbreitete bildgebende Verfahren sowie der geringeren Nachfrage von klinischen Ärzten nach Autopsien hat sich auch der Schwerpunkt der Pathologie im Laufe der Jahrzehnte stark verlagert. Hinzu kommen veränderte gesundheitspolitische und infrastrukturelle Rahmenbedingungen, die eine Anpassung der Obduktionspathologie an die Anforderungen der modernen Medizin erfordern. Die COVID-19-Pandemie hat die Bedeutung der Autopsie für ein besseres Verständnis von Krankheitsprozessen und die Entwicklung von präventiven und therapeutischen Konzepten unterstrichen und das Interesse verschiedener medizinischer Fachrichtungen an dem Potenzial wissenschaftlicher Erkenntnisse durch Autopsien wiederbelebt. So wurden im Rahmen des interdisziplinären deutschen Obduktionsnetzwerks DEFEAT PANDEMIcs/NATON (BMBF, 01KX2021 und 01KX2121) wichtige Erkenntnisse zur COVID-19-Erkrankung generiert [1] und die Grundlagen für fachübergreifende Konzepte geschaffen.

## Autopsie 2.0 oder weiter wie bisher?

An der Technischen Universität München (TUM) haben wir ein Konzept für postmortale Diagnostik und Forschung entwickelt, das die Fachbereiche Pathologie, klinische Bildgebung (Ultraschall, US) und Forschungseinrichtungen wie die Gewebebank des Klinikums Rechts der Isar integriert. Die Gewinnung von Gewebeproben von COVID-19-Verstorbenen erfolgt mittels ultraschallgesteuerter Nadelbiopsien nach standardisierten Verfahrensanweisungen [2]. Solche Verfahren sind in der Literatur als minimal-invasive Autopsie (MIA) oder postmortale minimal-invasive Gewebeprobenentnahme („minimally invasive tissue sampling“, MITS) bekannt. Dabei wird Gewebe ohne Eröffnung der Körperhöhlen in der Regel durch transkutane Nadelbiopsien gewonnen, oft unter Verwendung bildgebender Verfahren wie Ultraschall, Computertomographie oder Magnetresonanztomographie. Obwohl erste Versuche einer nichtinvasiven postmortalen Probenentnahme bereits im 19. Jahrhundert existierten, wurde diesem Ansatz in medizinisch hochentwickelten Ländern bisher wenig Aufmerksamkeit geschenkt. Veröffentlichte Studien stammen dementsprechend größtenteils aus Ländern mit begrenzten medizinischen Ressourcen [3, 4] und befassten sich hauptsächlich mit Infektionskrankheiten [5–7]. Das diagnostische Potenzial der nicht- oder minimal-invasiven Probenentnahme im Vergleich zur konventionellen Autopsie, insbesondere in Kombination mit bildgebenden Verfahren, wurde wiederholt gezeigt [8, 9].

Eine wichtige Voraussetzung für die Erzeugung valider Untersuchungsergebnisse an postmortalen Gewebeproben, insbesondere über die deskriptive Morphologie hinaus, ist der bestmögliche Erhaltungszustand der Gewebe. Das sog. postmortale Intervall (die Zeitspanne zwischen dem Tod und dem Beginn der Gewebekonservierung) und die Art der Konservierung haben einen erheblichen Einfluss auf die Qualität [10, 11]. Es ist daher wünschenswert, die Gewinnung und Konservierung von Gewebeproben so schnell wie möglich nach dem Tod eines Patienten durchzuführen. In den letzten Jahren wurden erfolgreich sogenannte „rapid research autopsies“ (RRA) eingeführt [12]. Die Durchführung erfordert erhebliche personelle und infrastrukturelle Ressourcen und wird daher weiterhin nur in speziellen wissenschaftlichen Kontexten eingesetzt. Auf Grundlage der bei uns erfolgreich etablierten interdisziplinären US-MIA präsentieren wir in diesem Beitrag eine Machbarkeitsstudie, in der wir die US-MIA kurz nach dem Tod von COVID-19-Verstorbenen am Krankenbett durchgeführt haben.

**Figure 1:**
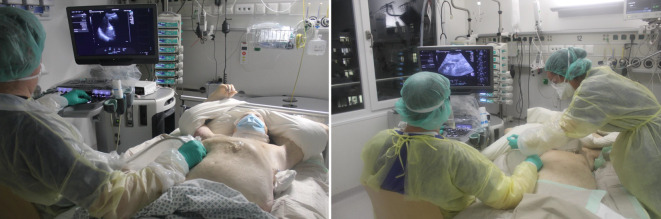

Das Ziel der Studie war die Bewertung der organisatorischen Durchführbarkeit, der zeitlichen Effizienz und des infektionshygienischen Risikos. Hierzu wurden die Zeitpunkte der einzelnen Verfahrensschritte dokumentiert (Tod, Einwilligung, Information des Teams, Beginn der Ultraschalluntersuchung, Beginn und Abschluss der Probenahme sowie Probenkonservierung). Nach Abschluss der US-MIA wurden virologische Abstriche von der persönlichen Schutzausrüstung der Beteiligten (Handschuhe, Schürze, Armstulpen) sowie von verschiedenen Oberflächen (Ultraschallsonde, Tastatur, Bildschirm, Ultraschallgelflasche, Arbeitsplatte) durchgeführt. Die gemäß Standardprotokoll gewonnenen Multiorganbiopsien wurden hinsichtlich ihrer Qualität (Vitalität und Länge der Biopsie) sowie lichtmikroskopisch erkennbarer pathologischer Veränderungen bewertet. Einschlusskriterien waren eine zu Lebzeiten bestätigte SARS-CoV-2-Infektion und die Zustimmung der nächsten Angehörigen. Nach Eintritt des Todes wurden die Angehörigen der COVID-19-Patienten telefonisch über das Verfahren informiert und um Zustimmung gebeten. Bei positivem Bescheid wurde umgehend das interdisziplinäre Einsatzteam informiert, bestehend aus einem fachärztlichen Internisten mit besonderer Ultraschallqualifikation (DEGUM III), einem Facharzt für Pathologie und einem technischen Mitarbeiter zur Unterstützung des Probenmanagements. Folgende Geräte und Instrumente wurden verwendet: Siemens Acuson S 3000-Ultraschallsystem (Siemens Medical Solutions, Mountain View, CA, USA), lineare 4‑bis-9-MHz- und konvexe 1‑bis-4-MHz-Schallköpfe, Nadelführungssystem (Ultra-Pro II; Civco, IA, USA), Einweg-Biopsiegerät (14 G, 20 cm Länge, 2,2 cm Hublänge; PlusSpeed®, Pflugbeil, Zorneding, Deutschland). Die Ultraschalluntersuchung und die Punktionen erfolgten bei Rückenlage des Verstorbenen nach standardisierten Protokollen ([13]; Abb. 1). Für jedes Organ oder jede Zielregion wurden 3 Proben entnommen und in 4 % gepuffertem Formalin, PAXgene Tissue FIX ((Qiagen, Hilden, Deutschland) fixiert oder bei −80 °C kryokonserviert. Die mikroskopische Qualitätskontrolle und diagnostische Bewertung erfolgte an FFPE-Geweben anhand von Hämatoxylin- und Eosin-gefärbten Schnittpräparaten sowie gegebenenfalls weiteren Standardfärbungen (PAS, Elastica van Gieson, Ladewig, Gomori und Chloracetat-Esterase).

**Figure 2:**
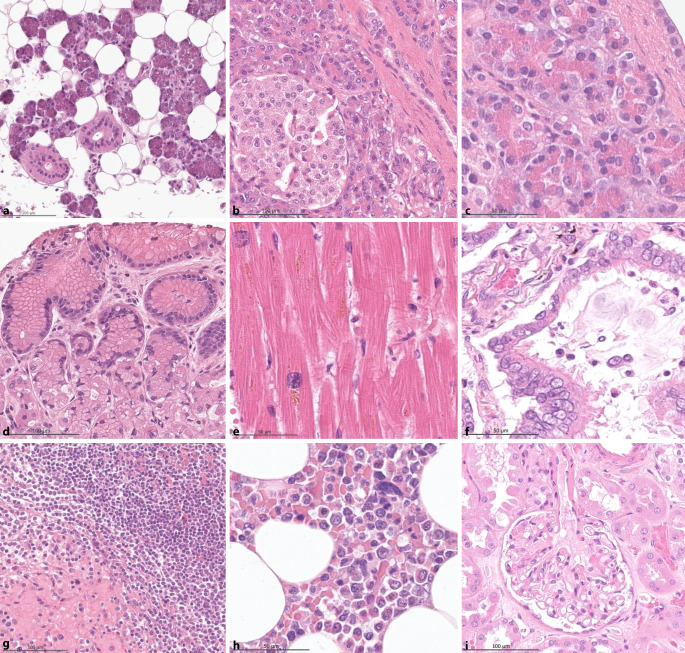

Zwischen Dezember 2021 und Januar 2022 verstarben 6 COVID-19-Patienten auf der Intensivstation. Bei 5 von 6 Fällen stimmten die Angehörigen der US-MIA zu. Diese wurden zwischen 129 und 210 min nach dem Tod durchgeführt, im Durchschnitt nach 162 min. Die Ultraschalluntersuchung dauerte durchschnittlich 13 min (5–16 min), die Punktionen und die Gewebegewinnung dauerten im Durchschnitt 54 min (44–75 min). Die Gesamtdauer der US-MIA lag somit im Durchschnitt bei 67 min, das postmortale Intervall betrug durchschnittlich 215 min. Insgesamt wurden 318 Multiorganproben gewonnen und 123 FFPE-Biopsien hinsichtlich ihrer Qualität und Diagnose bewertet. Von den Gewebeproben enthielten 96 % organspezifisches, diagnostisch verwertbares Gewebe. Der Erhaltungszustand der Gewebe war exzellent, mit lichtmikroskopisch und exemplarisch auch elektronenmikroskopisch nachgewiesenen optimal erhaltenen zellulären und subzellulären Strukturen (Abb. 2). Die durchschnittliche Biopsielänge betrug 11 mm (4–25 mm). In allen Lungenproben wurden lichtmikroskopisch typische SARS-CoV-2-induzierte Veränderungen in unterschiedlicher Ausprägung beobachtet, darunter ein diffuser Alveolarschaden in verschiedenen Stadien, Metaplasien, Mikrothromben, Endothelialitis und in einem Fall eine *Aspergillus*-Superinfektion. Extrapulmonale Befunde umfassten schockbedingte Veränderungen, insbesondere in der Leber und Niere, sowie verschiedene Grunderkrankungen. Die postmortale Ultraschalluntersuchung enthüllte pathologische Befunde an verschiedenen Organen, die vor dem Tod des Patienten nicht bekannt waren, darunter eine Pankreaszyste mit pankreaner intraepithelialer Neoplasie 1a, ein Niereninfarkt bei *Aspergillus*-Sepsis, ein frisches retroperitoneales Hämatom und eine sich organisierende Beinvenenthrombose. In Bezug auf die infektionshygienische Sicherheit des Verfahrens konnten wir erneut bestätigen [14], dass die US-MIA nicht zur Kontamination der Umgebung führt. Bei 54 von 55 auf SARS-CoV‑2 getesteten Abstrichen wurde keine virale SARS-CoV-2-RNA nachgewiesen. Lediglich in einem Abstrich (Ultraschallsonde mit unmittelbarem Kontakt zur Nadeleinstichstelle) konnte SARS-CoV-2-RNA nachgewiesen werden (CT/ORF 37).

## Die bettseitige US-MIA ist sicher und kann schnell nahezu autolysefreies Gewebe sichern

Angesichts der sinkenden Autopsieraten und der postmortalen Autolyse ist die Entwicklung neuer Konzepte für die postmortale Diagnostik und Forschung bei schweren Erkrankungen dringend erforderlich. Die sog. bettseitige minimal-invasive Autopsie (MIA) oder das „minimally-invasive tissue sampling“ (MITS) bieten vielversprechende interdisziplinäre Ansätze, um den aktuellen Herausforderungen der Obduktionspathologie und den veränderten Qualitätsanforderungen an das Gewebe zu begegnen. Obwohl die klinische Autopsie nach wie vor unverzichtbar ist und weiterhin als Goldstandard der fachübergreifenden Qualitätssicherung in der klinischen Medizin gilt, sollten moderne, fächerübergreifende Konzepte für die postmortale Diagnostik und Forschung verfolgt werden. Möglicherweise kann die Pathologie allein diese Aufgabe aufgrund der Veränderungen im Fachbereich nicht vollständig bewältigen. Die Interdisziplinarität und hohe Skalierbarkeit des hier vorgestellten Verfahrens bieten Chancen für eine erfolgreiche Modernisierung der Obduktionspathologie.

## Fazit für die Praxis

- Die postmortale ultraschallgesteuerte Nadelpunktion am Krankenbett ist zeiteffizient und infektionshygienisch sicher durchführbar und liefert nahezu autolysefreies Gewebe.
- Die ultraschallgesteuerte minimal-invasive Autopsie (US-MIA) könnte zukünftig eine geeignete Ergänzung zur konventionellen klinischen Autopsie darstellen und dazu beitragen, die postmortale Diagnostik in die Moderne der pathologischen Tiefenanalyse (Omics) zu führen.
- Der Erfolg der bettseitigen US-MIA beruht auf einer guten interdisziplinären Zusammenarbeit und der Nutzung vorhandener klinischer Ressourcen.
